# Case Report: Kaposiform hemangioendothelioma with PIK3CA mutation successfully treated with sirolimus

**DOI:** 10.3389/fonc.2023.1132702

**Published:** 2023-05-19

**Authors:** Zuopeng Wang, Hanlei Yan, Yangyang Ma, Wei Yao, Shan Zheng, Kai Li

**Affiliations:** ^1^ Department of Pediatric Surgery, Children’s Hospital of Fudan University, National Children’s Medical Center, Shanghai, China; ^2^ Department of Pathology, Children’s Hospital of Fudan University, National Children’s Medical Center, Shanghai, China

**Keywords:** PIK3CA, mutation, sirolimus, Kaposiform hemangioendothelioma, mTOR

## Abstract

Kaposiform hemangioendothelioma (KHE) is an extremely rare, locally aggressive vascular neoplasm. The etiopathogenesis of KHE is still poorly understood. In the present study, we found a new mutation in KHE (c.685delA, p.Thr229fs). The KHE patient with the PIK3CA mutation showed complete regression after sirolimus treatment. We propose that the presence of the PIK3CA mutation in KHE may correlate with good response to sirolimus.

## Introduction

Kaposiform hemangioendothelioma (KHE) is an extremely rare, locally aggressive vascular neoplasm resulting from abnormal angiogenesis and lymphangiogenesis during infancy or early childhood. It is commonly complicated by the occurrence of the Kasabach-Merritt phenomenon, which is characterized by the association of a rapidly growing vascular tumor, thrombocytopenia and consumptive coagulopathy (1). Recently, Carli et al. reported a patient with KHE with a phosphatidylinositol-4,5-bisphosphate 3-kinase catalytic subunit alpha (PIK3CA) mutation (c.323G > A, p.Arg108His) and suggested that KHE may be a subtype of PIK3CA-related overgrowth spectrum (PROS) (2). PROS is defined as a phenotypic spectrum of developmental disorders caused by activating variants of the PIK3CA gene (3). In the present study, we found a different PIK3CA mutation in KHE (c.685delA, p.Thr229fs), which provides new evidence that KHE may belongs to PROS.

## Case report

**Figure 1:**
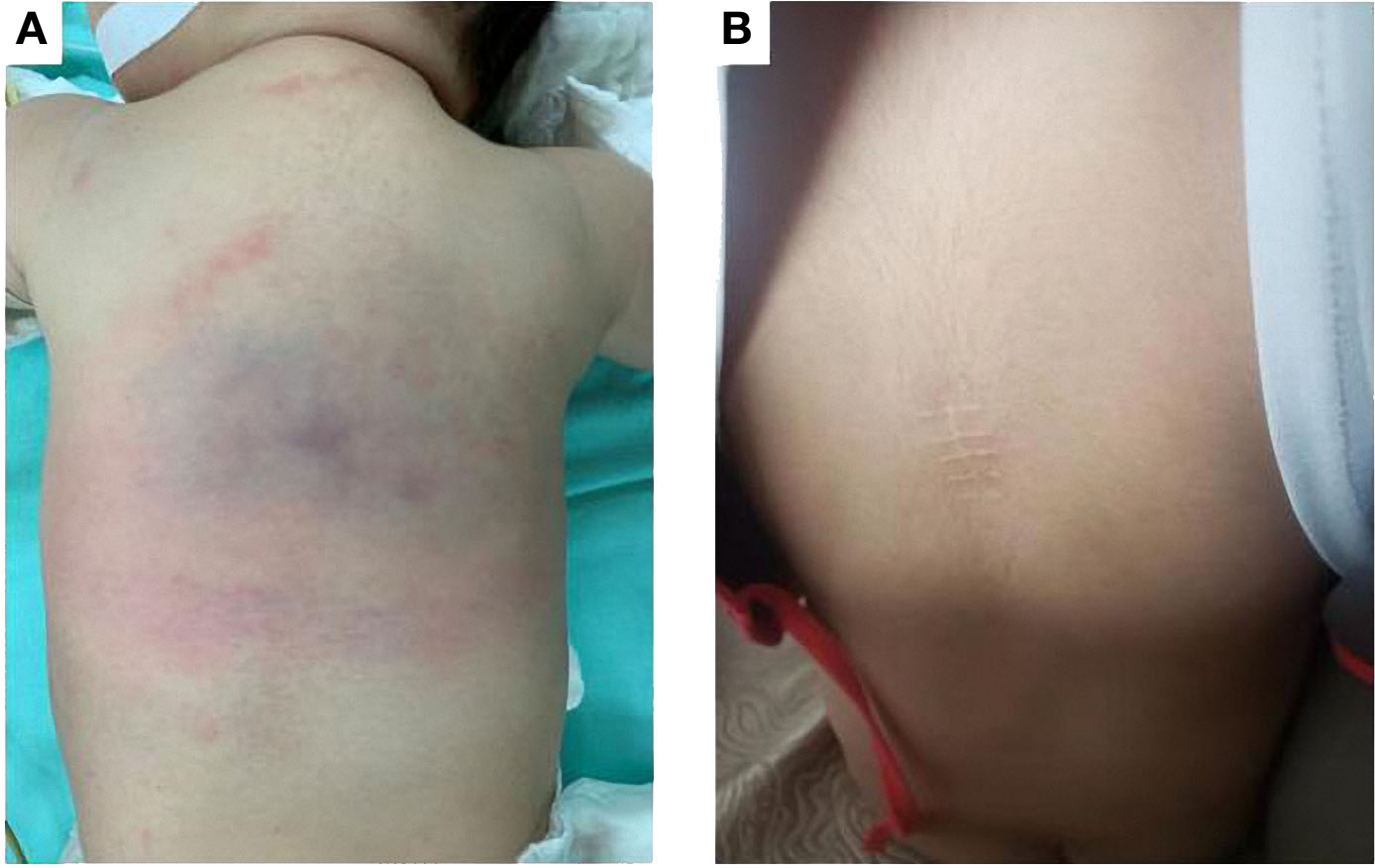
**(A)** Before treatment, an erythematous, poorly demarcated lesion (6 cm x 5 cm) on the back. **(B)** After two years of sirolimus treatment, the lesion regressed completely.

A 3-month-old boy was referred to Children’s hospital of Fudan university with a middorsal tumor growing for more than one month, which was misdiagnosed as hemangioma and received no treatment by the local hospital. Physical examination revealed an erythematous, poorly demarcated lesion (6 cm × 5 cm) on the patient’s back with no signs of inflammation or pain (**Figure 1A**). The patient had no family history of vascular disorders.

Laboratory examination found no obvious abnormality in routine blood and coagulation tests. Complete blood count at presentation revealed a platelet count of 302 × 10^9^/L (normal range:188-472× 10^9^/L) and hemoglobin level of 13.8 g/dl(normal range:11.2-14.9 g/dl). Coagulation function was normal including fibrinogen 209 mg/dl(normal range: 200-400 mg/dl), prothrombin time 13.3 s(normal range:11-14.5s), partial thromboplastin time 34 s (normal range:28-45s)and D-dimer level 1.99 mg/L (normal range: 0-0.5 mg/L).

**Figure 2:**
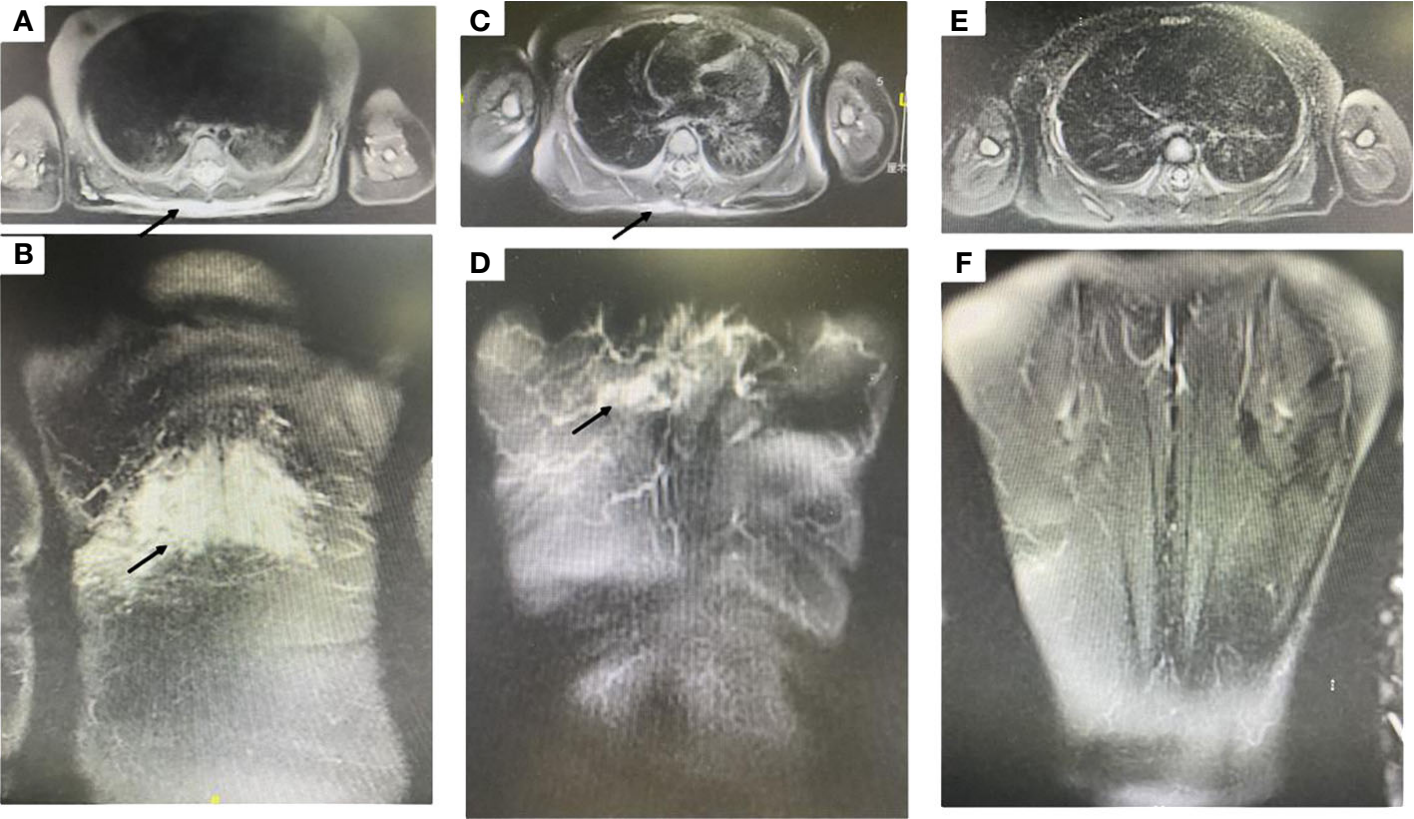
**(A, B)** Magnetic resonance imaging showed ill-defined margins, diffusive hyperintense signals on fat-suppressed T2 weighted images (black arrow designates the lesion). **(C, D)** After one year of sirolimus treatment, the lesion shrank dramatically (black arrow designates the lesion). **(E, F)** After two years of sirolimus treatment, the lesion regressed completely.

Magnetic resonance imaging showed ill-defined margins and diffusive hyperintense signals in fat-suppressed T2 weighted images (**Figures 2A, B**) and obvious heterogeneous enhancement in T1 weighted images.

**Figure 3:**
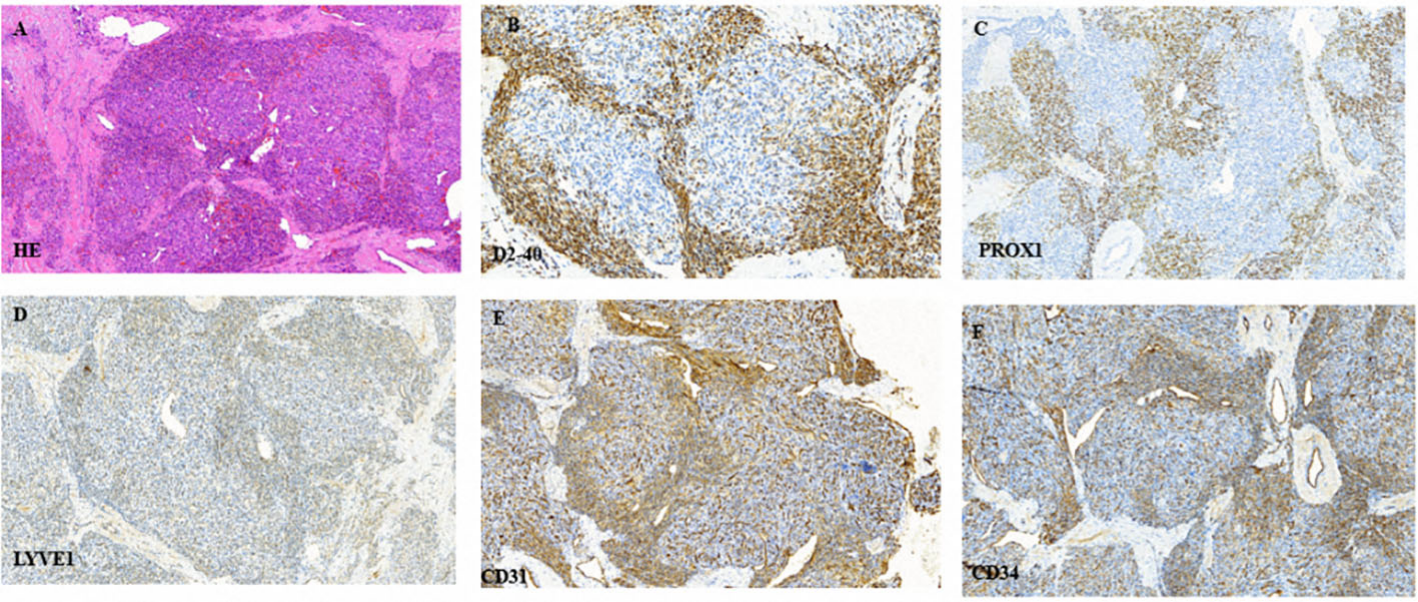
**(A)** Typical glomeruloid areas with spindle shaped cells following hematoxylin and eosin staining. **(B–F)** Immunohistochemical staining was positive for D2–40, PROX-1, LYVE-1, CD31 and CD34, respectively.

The patient underwent biopsy that confirmed the pathological diagnosis of KHE. Hematoxylin and eosin staining showed typical glomeruloid areas with spindle shaped cells. Immunohistochemical staining was positive for D2–40, PROX-1, LYVE-1, CD31 and CD34 (**Figure 3**). We also detected the extracted DNA in tumor tissue samples from biopsy by next generation sequencing and bioinformatics analysis. We focused on hot-spot mutation regions of 42 genes (**Table 1**) related to tumorigenesis and vascular diseases and found that the patient harbored PIK3CA mosaic pathogenic variants (c.685delA and p.Thr229fs).

**Table 1:** Panel of 42 genes with mutations related to tumorigenesis and vascular diseases.

ABL1	ACVRL1	AKT1	ALK	BRAF	BRCA1	BRCA2
EGFR	ENG	EPHB4	ERBB2	FGFR2	FGFR3	FLT3
FLT4	GLMN	GNA11	GNA14	GNAQ	HRAS	IDH1
IDH2	KDR	KIT	KRAS	MAP2K1	MAP3K3	MAPK1
MAPK3	MET	mTOR	NRAS	PDGFRA	PDGFRB	PIK3CA
PTEN	RASA1	RET	SMAD4	STAMBP	TEK	TSC1

The patient received sirolimus therapy following diagnosis of KHE without the Kasabach-Merritt phenomenon. We administered the sirolimus regimen at an initial dose of 0.8 mg/m^2^, twice a day and maintained a drug trough level of 10-15 ng/mL (4). Sulfamethoxazole was then applied for prophylaxis of pneumocystis carinii pneumonia. The lesion shrank dramatically after one year of sirolimus therapy (**Figures 2C, D**). The patient achieved complete response after two years of sirolimus treatment (**Figures 1B**, **2E, F**). The lesion showed total regression, and no serious complications were observed.

## Discussion

The etiopathogenesis of KHE is still poorly understood. Sporadic vascular tumors have been documented with potential genetic anomalies. Most mutations are detected in genes that play crucial roles in pathways involved in angiogenesis and lymphangiogenesis, vascular cell growth, apoptosis and proliferation (5). There are no treatment guidelines for KHE, although there are some expert consensus (6) and treatment recommendations (7). Sirolimus (8), vincristine (9) and glucocorticoids (6) are the common medication regimens for KHE.

A few mutations and genes had been found to be related to the development of KHE. Zhou et al. demonstrated a somatic translocation between chromosomes 13 and 16 at the bands of 13q14 and 16p13.3 in 10% of cells with KHE nodules; normal cells were also present in the karyotype (10). Lim et al. identified a single heterozygous somatic single nucleotide variation, c.614A>T (p.Gln205Leu), in GNA14 (G-protein subunit alpha 14) in one of three KHE patients (11). They subsequently confirmed that somatic activation of the GNA14 mutation caused changes in cellular morphology and rendered cells growth-factor independent by upregulating the mitogen-activated protein kinase (MAPK) pathway. A recent study also revealed that PIK3CA mutations correlated with mammalian target of rapamycin (mTOR) pathway expression (12), but not with clinical or pathological features, in patients with fibro-adipose vascular anomaly (13), indicating that sirolimus may still be effective in patients without PIK3CA mutations. Sirolimus inhibits the mTOR pathway, with subsequent effects on angiogenesis and lymphangiogenesis. Maruani et al. reported that inhibiting mTOR may help shrink lesions and improve clinical symptoms associated with lymphatic anomalies, even with no evidence of PIK3CA variants (14). We analyzed the expression of mTOR-related proteins in KHE and found that the absence of tuberous sclerosis complex 2 and phosphatase and tensin homolog caused abnormal activation of the mTOR signaling pathway and may account for the pathogenesis of KHE (15). In our study, the patient with a new PIK3CA mutation showed complete response to sirolimus therapy. The PIK3CA mutation may have abnormally activated the mTOR pathway, although the pathogenicity of this mutation has not been confirmed. In our next study, we will construct the mutant gene in zebrafish to observe the phenotype of the PIK3CA mutation.

The patient is still in maintenance treatment using a lower concentration of sirolimus. At present the standard for sirolimus withdrawal is still controversial, since the rebound of KHE after sirolimus treatment has been reported in approximately 17% of patients (16). Additionally, Venot et al. provided the first direct evidence supporting PIK3CA inhibition as a promising therapeutic strategy in patients with PROS (17) by inhibiting the constitutively activated p110α subunit of PI3K using targeted molecules, such as BYL719 (Alpelisib) (18). They also proposed new ideas for the treatment of KHE, especially for KHE patients with PIK3CA mutations that show a poor response to existing treatment options.

In conclusion, we have presented a new PIK3CA mutation (c.685delA, p.Thr229fs) in KHE. KHE with this PIK3CA mutation may be correlated with a good response to sirolimus. However, more cases are required to validate our findings.

## Data availability statement

The datasets presented in this study can be found in online repositories. The names of the repository/repositories and accession number(s) can be found in the article/**Supplementary Material**.

## Ethics statement

Written informed consent was obtained from the patient’s parents for the publication of any potentially identifiable images or data included in this article.

## Author contributions

KL and ZW conceived and designed the study. ZW, HY, WY and SZ collected the clinical data and performed data analysis. SZ and YM offered the assist in data collection. ZW, HY and KL wrote the paper. KL, SZ and ZW reviewed and edited the manuscript. All authors read and approved the manuscript.
